# Single modality radical radiotherapy is an acceptable alternative for the older patient with squamous cell carcinoma of the oesophagus

**DOI:** 10.1136/bmjgast-2020-000492

**Published:** 2021-01-27

**Authors:** Sarah Derby, Matthew Forshaw, Caroline Lowrie, Derek Grose, Husam Marashi, Philip McLoone, Christina Wilson, David McIntosh

**Affiliations:** 1 Clinical Oncology, Beatson West of Scotland Cancer Centre, Glasgow, UK; 2 Institute of Cancer Sciences, University of Glasgow, Glasgow, UK; 3 Upper Gastrointestinal Surgery, Glasgow Royal Infirmary, Glasgow, UK; 4 Biostatistics, University of Glasgow Institute of Health and Wellbeing, Glasgow, UK

**Keywords:** oesophageal cancer, radiation therapy, cancer, chemotherapy

## Abstract

**Background:**

Oesophageal cancer remains a common cause of cancer mortality worldwide. Increasingly, oncology centres are treating an older population and comorbidities may preclude multimodality treatment with chemoradiotherapy (CRT). We review outcomes of radical radiotherapy (RT) in an older population treating squamous cell carcinoma (SCC) oesophagus.

**Methods:**

Patients over 65 years receiving RT for SCC oesophagus between 2013 and 2016 in the West of Scotland were identified. Kaplan-Meier and Cox-regression analysis were used to compare overall survival (OS) between patients treated with radical RT and radical CRT.

**Results:**

There were 83 patients over 65 years treated with either RT (n=21) or CRT (n=62). There was no significant difference in median OS between CRT versus RT (26.8 months vs 28.5 months, p=0.92). All patients receiving RT completed their treatment whereas 11% of CRT patients did not complete treatment.

**Conclusion:**

Survival in this non-trial older patient group managed with CRT is comparable to that reported in previous trials. RT shows better than expected outcomes which may reflect developments in RT technique. This review supports RT as an alternative in older patients, unfit for concurrent treatment.

**Summary box**

**What is already known about this subject?**

- Chemoradiotherapy (CRT) in squamous cell carcinoma (SCC) oesophagus is an effective radical treatment; however, older populations may not be fit for radical CRT.

**What are the new findings?**

- Radiotherapy (RT) can have comparable survival in older patients, unfit for concurrent chemotherapy.

**How might it impact on clinical practice in the foreseeable future?**

- RT should be considered as an acceptable alternative for SCC oesophagus in those unfit for chemotherapy.

## Background

Oesophageal cancer is one of the most common causes of cancer death in the UK and worldwide.^1 2^ Radiotherapy (RT), with or without concurrent chemotherapy is an option for radical management in appropriate patients.^1–3^ With an ageing population it is increasingly common to offer a radical approach to the older patient. Over 40% of new cases are now patients aged 75 years or over.^4^ Squamous cell carcinoma (SCC) of the oesophagus is associated with risk factors such as smoking and alcohol and is less common than adenocarcinoma of the oesophagus which is increasing in incidence. Management between adenocarcinoma and SCC of the oesophagus can differ. Non-surgical treatment with radical RT or chemoradiotherapy (CRT) in SCC is accepted as an appropriate treatment strategy.^5–8^ While the incidence of squamous carcinoma of the oesophagus has been declining in recent years, it remains an important proportion of the population referred for treatment. Worldwide, SCC still accounts for 90% of oesophageal cancers; however, western countries have shown decline in recent years such as in the USA. Between 1975 and 2001 SCC oesophagus dropped from 31 per million to 19 per million whereas adenocarcinoma conversely increased from 3.8 to 23.3 per million in the same timeframe.^9 10^ As the population of patients with cancer ages there will be an increasing challenge in managing older patients with SCC oesophagus.^11 12^

We report the experience of a large tertiary cancer centre treating SCC of the oesophagus with RT alone as an alternative in those patients not deemed fit for concurrent chemotherapy or where chemotherapy is contraindicated due to performance status (PS), comorbidity or patient preference. This centre has previously published favourable data on outcomes of radical RT for SCC oesophagus.^13^ Here, a review of the over 65 population is specifically addressed.

## Methods

### Study design

**Figure 1:**
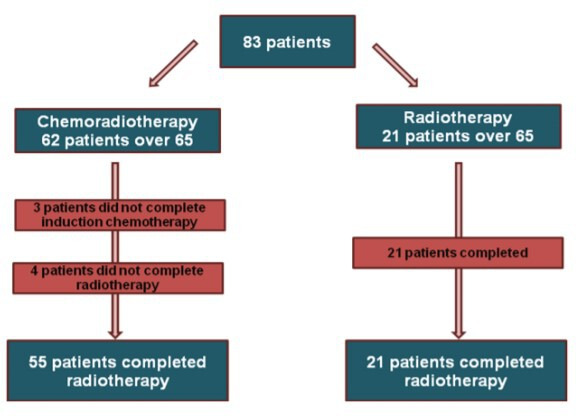
An outline of patient selection and completion of treatment for all patients treated between 2013 and 2016.

This was a retrospective review of patients with SCC who were radically treated between March 2013 and March 2016 in the Beatson West of Scotland Cancer Centre, looking at PS, tumour, node, metastases (TNM) 7 staging, tumour length and treatment length along with survival of patients.^14^ Patients underwent endoscopic ultrasound (EUS), CT and positron emission tomography (PET) as part of tumour staging. Data were collected using electronic patient records and appropriate ethical permissions were obtained. Patients were analysed on an intention to treat basis (see figure 1). Tumour length was measured from ECLIPSE RT planning software using the gross tumour volume (GTV) from peer-reviewed RT plans. All plans were peer-reviewed at the volume assessment meeting with between two and five site-specialist consultants assessing each volume. The study team chose to use GTV length as a surrogate for tumour length as it was available for all patients. The tumour length was not always clearly documented from the diagnostic imaging or EUS, particularly in impassable tumours. EUS report, which traditionally includes anatomical landmarks and tumour length, as well as PET were central to the delineation of GTV contouring.

#### Treatment

There were 83 patients aged over 65 years identified: 21 received radical RT compared with 62 patients who had CRT during this period. Patients identified as receiving CRT had chemotherapy concurrently prescribed. The chemotherapy regimen used in all patients was cisplatin/5FU with an initial induction cycle followed by two concurrent cycles. Standard fractionation dose for the RT only group was the accelerated, hypofractionated 55 Gy in 20 fractions (RT 19/21 patients: other doses 52 Gy/20#, 50 Gy/25#). Standard RT fractionation for CRT patients was 50 Gy in 25 fractions (CRT 60/62 patients: other doses 54 Gy/27#, 55 Gy/20# prescribed). Standard of care for SCC oesophagus in this centre is CRT and surgery is only offered as a potential salvage option for persistent or relapsed disease.

### RT planning

RT in this centre is delivered using intensity-modulated RT (IMRT) and more recently volumetric modulated arc therapy (VMAT) which can allow for improved conformality of RT dose and lower dose to organs at risk (OARs). Planning for these patients was a mix of IMRT 3 and 4 field RT planning with migration to a primarily VMAT solution in 2015. RT GTV was defined using composite information provided from EUS, CT and PET imaging. GTV was recorded from plans as a surrogate for tumour length in all patients. SCOPE-1 RT protocol was used as standard for delineation of clinical target volumes and planning target volumes.^15^

#### Statistical analysis

The characteristics of patients receiving RT or CRT were summarised using medians or percentages as appropriate and mean in reporting age. Differences were identified using Wilcoxon rank-sum test or Pearson’s χ^2^ test. Survival was calculated from date of diagnosis until date of death or censor date (September 2018). Overall survival (OS) was estimated using the Kaplan-Meier method. Univariable and multivariable analysis of differences in survival between patients classified by each of sex, Eastern Cooperative Oncology Group (ECOG) PS, stage, modality and GTV length were performed using log-rank tests and Cox-proportional hazards regression. All tests were two-sided and a p value <0.05 was considered statistically significant. Analyses were carried out using STATA V.14 (Statacorp).

## Results

### Patient characteristics

A total of 83 patients were included in the study (RT n=21, CRT n=62, see table 1). There was a greater percentage of female patients in the RT group compared with the CRT group (RT 66.7% vs CRT 48.4%, see table 1). There was also a higher percentage of patients over 75 years old within the RT group (RT 80.9% vs CRT 33.9%). In the RT group, 33.3% of patients were PS 0 and in the CRT group 51.6% patients were PS 0. T and N staging was similar in both groups as was overall stage.

**Table 1:** Patient characteristics

Characteristic	No. of patients (%)	Chemoradiotherapy (n=62)	Radiotherapy (n=21)	P value
Patient details	All patients (n=83)			
Age (years)				
Mean (SD; range)	74.3 (±5.36; 65–86)	72.8 (±4.22; 65–83)	79.0 (±5.71; 66–86)	p=0.0001 p≤0.0001
>75 (%)	38 (45.8)	21 (33.9)	17 (80.9)	
Gender				
Male	39 (47.0)	32 (51.6)	7 (33.3)	p=0.147
Female	44 (53.0)	30 (48.4)	14 (66.7)	
ECOG performance status (PS)				
PS 0	39 (47.0)	32 (51.6)	7 (33.3)	p=0.001
PS 1	37 (44.6)	29 (46.8)	8 (38.1)	
PS 2	7 (8.4)	1 (1.61)	6 (28.6)	
Overall stage TNM 8				
Stage I	6 (7.2)	5 (8.1)	1 (4.8)	p=0.485
Stage II	45 (54.2)	31 (50.0)	14 (66.7)	
Stage III	30 (36.14)	24 (38.71)	6 (28.57)	
Unknown	2 (2.4)	2 (3.2)	–	
GTV length				
Median (cm) (SD; IQR)	5.7 (±3.01; 4.1–8.25)	6.2 (±3.28; 4.1–8.8)	5.2 (±1.94; 4.1–6.5)	p=0.281
≤6 cm	41 (49.4)	29 (46.8)	12 (57.1)	
>6 cm	39 (46.99)	30 (48.8)*	9 (42.90)	p=0.532

Summary of patient characteristics, overall stage: by TNM 8 definition.

*Three GTV values unavailable in chemoradiotherapy group.

GTV, gross tumour volume; TNM, tumour, node, metastases.

### Compliance

Over 95% (n=60) of CRT patients were prescribed 50 Gy in 25 fractions and 90.5% (n=19) of RT patients were prescribed 55 Gy in 20 fractions.

There were seven patients in the CRT group that did not complete RT consisting of three patients that did not complete induction chemotherapy and so did not proceed to RT and four patients in the CRT group that started RT but did not complete the treatment course (figure 1). Reasons for CRT non-completion included oesophageal perforation, stroke and deterioration in PS. All 21 patients in the RT group completed treatment.

### Tumour lengths

PET and EUS reports were used as part of the GTV RT planning process. Length of tumour was identified based on the length stated on either EUS, endoscopy or radiology reports. However, there were 18 patients that did not have a recorded length based on these investigations. The main reason for the missing data was the presence of impassable disease recorded on EUS in 11 patients. This resulted in the absence of a lower border being reported (CRT=9, RT=2). Therefore, GTV length identified on RT planning software was used as an alternative comparative marker to reported EUS length as a surrogate length across all patients to provide a standardised approach in measurement.

### Survival

**Figure 2:**
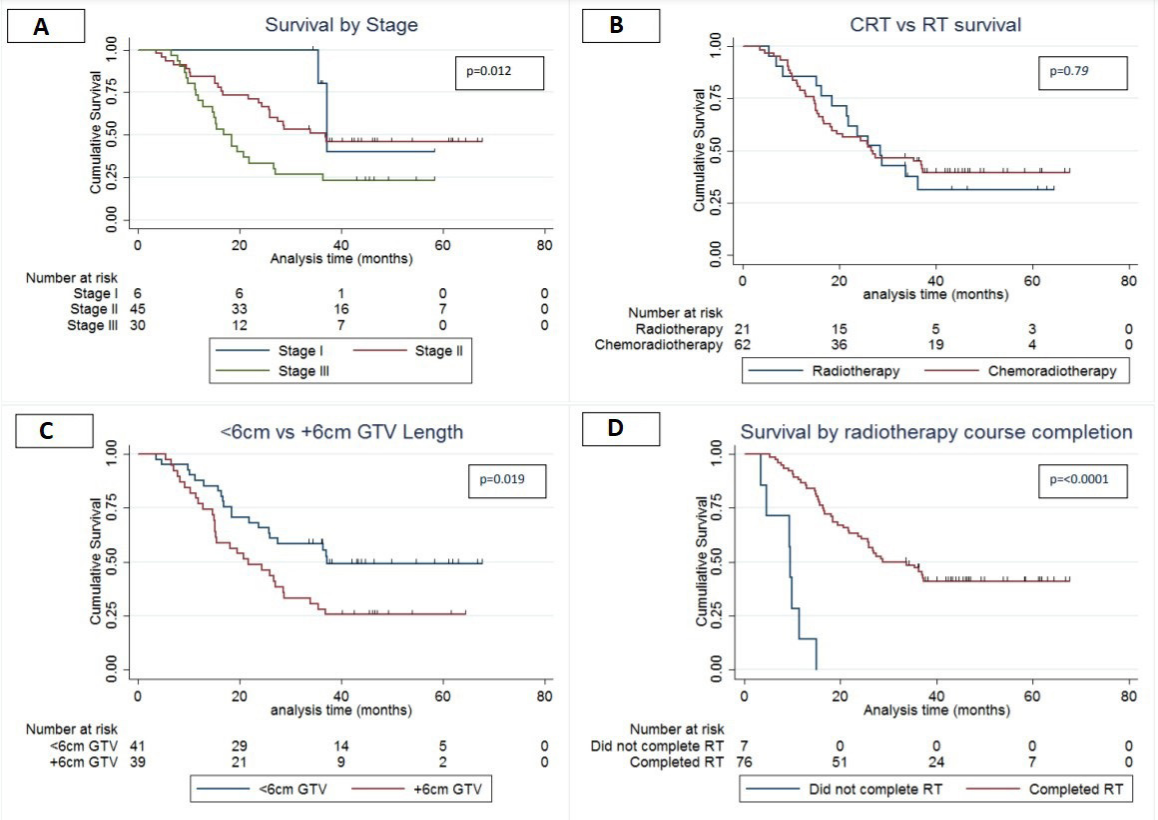
(A) Median overall survival (OS) stage I—37.3 months (95% CI 35.5 to na), stage II—37.0 months (95% CI 25.7 to na), stage III—16.8 months (95% CI 12.83 to 26.7); p=0.012. (B) Median OS radiotherapy (RT): 28.5 months (95% CI 20.7 to 37.1), chemoradiotherapy (CRT): 26.8 months (95% CI 16.8 to na); p=0.79. (C) Median OS <6cm: 37.3 months (95% CI 23.8 to na), ≥6 cm: 21.6 months (95% CI 15.1 to 28.8); p=0.019. (D) Median OS completed treatment=28.8 months (95% CI 23.8 to na), did not complete treatment=9.53 months (95% CI 3.53 to 11.4); p≤0.0001; na=unable to calculate CI limit. GTV, gross tumour volume.

Median OS for all patients was 27.0 months (95% CI 20.7 to 37.1) calculated on an intention to treat basis. When divided into cancer stage, patients with early stage cancers had better survival than late stage cancers (stage I=37.3 months; 95% CI 35.5 to na vs stage III=16.8 months; 95% CI 12.83 to 26.7; p=0.02, see figure 2A). However, there was no statistically significant difference in median OS between the RT and CRT group (RT 28.5 months 95% CI 16.8 to na, CRT 26.7 months; 95% CI 18.4 to na, p=0.79, see figure 2B). There was a non-significant increased risk of death in the male population versus females (male: HR 1.17; 95% CI 0.66 to 2.10; p=0.574). Patients with GTV lengths of ≥6 cm had a greater risk of death irrespective of treatment modality (HR for ≥6 cm—1.86, p=0.041, see figure 2C, table 2). Patients with a PS of ≥1 and stage III disease were also associated with poorer outcomes, though stage III disease was non-significant in multivariable analysis (HR for PS≥1–2.11, p=0.020, HR for stage III disease—3.18, p=0.126, see figure 2A, table 2).

**Table 2:** Hazard of death: multivariable analysis

Patient characteristics	Unadjusted HR	95% CI	P value	Fully adjusted HR	95% CI	P value
Female	1			1		
Male	1.13	0.65 to 1.96	p=0.656	1.17	0.66 to 2.10	p=0.574
PS 0	1			1		
PS≥1	2.00	1.14 to 3.53	p=0.016	2.11	1.12 to 3.97	p=0.020
Stage I	1			1		
Stage II	2.07	0.49 to 8.79	p=0.326	1.55	0.35 to 6.79	p=0.563
Stage III	4.31	1.01 to 18.35	p=0.050	3.18	0.72 to 14.00	p=0.126
Radiotherapy	1			1		
Chemoradiotherapy	0.92	0.50 to 1.71	p=0.793	0.88	0.45 to 1.72	p=0.708
GTV<6 cm	1			1		
GTV≥6 cm	1.96	11.0 to 3.47	p=0.021	1.86	1.03 to 3.37	p=0.041

HRs by patient characteristics, treatment modality and gross tumour volume lengths.

GTV, gross tumour volume; PS, performance status.

Patients who did not complete planned RT had significantly poorer survival than patients who completed treatment (9.5 months vs 28.8 months figure 2D, p≤0.0001). Mean age for patients completing treatment and not completing treatment was 74.1 (SD ±4.34; range 66.7–77.9) and 74.4 (SD ±5.47; range 65.5–86.2).

## Discussion

In this retrospective study focussing on data from 2013 to 2016 in the Beatson West of Scotland Cancer Centre we found that most of the RT group were over 75 years (80.9%) compared with a significantly younger CRT group (mean age=72.8 years, percentage over 75=33.9%, p=0.0001). The difference in survival between sexes was non-significant although trended towards an increased risk of death in the male population. This may warrant future investigation in the context of the higher percentage of females in the RT group. Though the numbers are relatively small, this higher percentage should also be considered when looking at the relatively good survival of the RT group as improved survival in older women has been seen in other cancers such in lung cancer.^16–19^ There was no significant difference in median OS between CRT and RT groups (figure 2B). CRT median OS remains comparable with other centres and the SCOPE-1 data, which is reassuring on considering that this is an older, real world—population.^20–22^ As expected, poorer PS and longer tumours were all associated with poorer survival.

It is of interest that as part of this retrospective review that there is a disparity in ages between the CRT and RT group. Multiple clinical trials across numerous tumour types have demonstrated the risk of increased toxicity of combining chemotherapy with RT.^23–26^ The stark difference in age between RT and CRT groups may arise from a clinical concern about fitness for multimodality treatment as the older patient, while fit, may be physiologically frailer than a younger individual. This is often gestalt rather than based on objective measures. Geriatric assessment tools are an emerging field that will be important in determining objective rather than subjective assessment for suitability of concurrent treatment.^27 28^ Similarly, future clinical trials should aim to include older patients with robust assessment tools particularly as this particular subgroup is often underrepresented but is increasing in clinical relevance.^29^ This represents a larger discussion that remains ongoing in the oncology community when consider how to balance these issues where older patients have radically treatable cancers.

The survival of patients undergoing single modality RT is perhaps surprising given the historically low expectations for single modality RT patients.^24^ There may be several possible reasons for this. Historical RT fractionation was 64 Gy in 32 fractions compared with the hypofractionated dose of 55 Gy in 20 fractions used in this centre.^3 13 24^ It may be that this is a biologically superior dose as the 4-week period of treatment may offset the risk of cell repopulation after 28 days. Survival in this review is comparable to other centres using hypofractionated single modality RT and shows favourable outcomes in this patient group.^30^ New treatment modalities such as VMAT allow for excellent dose homogeneity to tumours with effective tissue simulation to estimate doses to primary tumour and OARs. Finally, another consideration is that this may also be an indication of appropriate patient selection and a pragmatic approach to frailer patients. It appears that single modality RT may be safe and deliverable in an older population.

There were several impassable tumours in the CRT group (n=9) which were included in the unrecorded tumour lengths. Of the 11 patients who had confirmed impassable tumours there were no long-term survivors after 3 years of follow-up. This may suggest that an impassable tumour on EUS or endoscopy is a negative prognostic indicator of outcome. Luminal tumour bulk is qualitatively recorded on investigation reports and it may be a factor that warrants future consideration.

Finally, in this cohort single modality RT was better tolerated compared with CRT. Completing treatment is an important priority for these patients and is reflected in the poorer outcomes of those not completing treatment in the CRT group. All patients in the RT group completed treatment compared with an 11% non-completion in the CRT group. This is comparable to non-completion rate in other centres and the SCOPE-1 trial which was around 10%.^13 20 22^ Palliative locally advanced oesophageal cancer survival is often challenging to estimate but may be expected to be between 6 and 9 months though not usually over a year.^31^ The survival of RT patients in this review certainly appears favourable compared with these palliative patients; however, the heterogeneity in these groups prevents direct comparison.

The major limitation is the retrospective nature of the study. Though this information can be applied more generally, the lack of prospective fitness assessment prohibits a more detailed assessment of this population’s characteristics. PS is a relatively limited descriptor and does not holistically represent true patient fitness.^27^ Future prospective reviews should use comprehensive geriatric assessment tools which give fuller assessments of this population as these patients are often heterogeneous in fitness and comorbidity.^32^ Similarly, toxicity data were not collected for these patients and can bring a well-rounded view on patient outcomes both on treatment and beyond. This is also an important focus for future reviews, particularly in older patient groups where quality of life rather than length of survival may be of greater priority. Furthermore, another limitation is that we have used GTV rather than prospectively gathered data from EUS, PET and CT to estimate tumour length. PET and EUS reports are integral to RT planning and are a key aspect which must inform GTV contouring.^33^ We feel that using GTV still provides a fair estimate of tumour length and our robust peer review process aims to maintain consistency.

## Conclusions

Data reviewed in this series confers comparable results to other centres with improvement on survival in single modality RT compared with results from historical data. Other tertiary cancer centres have also found that hypofractionated regimens can have favourable outcomes in patients not suitable for CRT. CRT should remain a standard of care in radical treatment of squamous oesophageal cancer in fit patients. This review supports the use of single modality RT as an alternative option for those who are not suitable for concurrent treatment and may allow a survival advantage over best supportive care in an older population.
